# Clinic vs. daily life gait characteristics in patients with spinocerebellar ataxia

**DOI:** 10.3389/fdgth.2025.1590150

**Published:** 2025-09-03

**Authors:** Vrutangkumar V. Shah, Daniel Muzyka, Adam Jagodinsky, Hannah Casey, James McNames, Mahmoud El-Gohary, Kristen Sowalsky, Delaram Safarpour, Patricia Carlson-Kuhta, Fay B. Horak, Christopher M. Gomez

**Affiliations:** ^1^ APDM Wearable Technologies—a Clario Company, Portland, OR, United States; ^2^ Department of Neurology, Oregon Health & Science University, Portland, OR, United States; ^3^ Department of Neurology, The University of Chicago, Chicago, IL, United States; ^4^ Department of Electrical and Computer Engineering, Portland State University, Portland, OR, United States

**Keywords:** gait, IMUs, wearable technology, daily life monitoring, spinocerebellar ataxia

## Abstract

**Background:**

Recent findings suggest that a single gait assessment in a clinic may not reflect everyday mobility.

**Objective:**

We compared gait measures that best differentiated individuals with spinocerebellar ataxia (SCA) from age-matched healthy controls (HC) during a supervised gait test in the clinic vs. a week of unsupervised gait during daily life.

**Methods:**

Twenty-six individuals with SCA types 1, 2, 3, and 6, and 13 (HC) wore three Opal inertial sensors (on both feet and lower back) during a 2-minute walk in the clinic and for seven days in daily life. Seventeen gait measures were analyzed to investigate the group differences using Mann–Whitney *U*-tests and area under the curve (AUC).

**Results:**

Ten gait measures were significantly worse in SCA than HC for the clinic test (*p* < 0.003), but only 3 were worse in daily life (*p* < 0.003). Only a few gait measures consistently discriminated groups in both environments. Specifically, variability in Swing Time and Double Support Time had AUCs of 0.99 (*p* < 0.0001) and 0.96 (*p* < 0.0001) in the clinic, and 0.84 (*p* < 0.0003) and 0.80 (*p* < 0.002) in daily life, respectively. Clinical gait measures showed stronger correlations with clinical outcomes (ie, SARA and FARS-ADL; r = 0.50–0.77) than between daily life gait measures (r = 0.31–0.49). Gait activity in daily life was not statistically significant between the SCA and HC groups (*p* > 0.06).

**Conclusions:**

Digital gait measures discriminate SCA in both environments. In-clinic measures are more sensitive, while daily life measures provide ecological validity, highlighting a trade-off and offering complementary insights.

## Introduction

Gait impairment is an early sign of spinocerebellar ataxia (SCA) that increases in severity with disease progression (1–3). Moreover, gait and balance impairments are among the most debilitating impairments exhibited with SCA, with deleterious impacts on daily function, fall risk, and quality of life (4). In clinical trials of ataxia, disease severity and progression are most commonly assessed using the Scale for Assessment and Rating of Ataxia (SARA) (5), which categorizes the severity of gait, balance, and other motor impairments on an ordinal scale. However, the SARA has several limitations, including high variance and subjectivity in scoring, low sensitivity, the need for large sample sizes in clinical trials, and the need for clinical specialists to administer testing (6–8). Given these limitations, there is a need for a quantitative assessment of SCA gait to evaluate therapeutic efficacy in both the clinic and daily living environments.

Advancements in wearable technologies have overcome financial and logistical limitations that have hindered the use of quantitative movement assessment in clinical trials, allowing for objective ataxic movements and gait measurement that is feasible in both clinical and daily life settings (9–26). Ilg et al. (11) found that gait variability measures captured in the laboratory and at home were able to discriminate between patients with cerebellar ataxia from healthy controls. Similar gait variability measures have been reported in laboratory gait assessment of prodromal and manifest SCA using wearable sensors (16, 21). Additionally, recent longitudinal studies indicate that gait variability may show sensitivity to progression in cerebellar ataxia patients, and that clinical trial sample size may be significantly reduced with the implementation of wearable sensors to capture accurate and objective measures reflective of motor symptom progression in SCA (27, 28). Daily life monitoring outside the clinic may be particularly useful as limited access to clinical specialists makes in-clinic assessment challenging. Furthermore, daily monitoring of gait using wearables in the home environment offers a comprehensive and real-life view of disease severity (11, 29).

Despite increasing adaptation of wearable technologies in SCA, relatively few studies have investigated whether the most discriminative gait features identified in clinical settings remain the same in real-world daily life settings. For example, Iig et al. (11) found that gait variability measures such as lateral step deviation and composite score (lateral step deviation and stride length variability) were statistically significant between cerebellar ataxia and healthy subjects in both in-clinic and real-life daily walking conditions, with higher effect sizes observed in the clinic settings. Similarly, Seemann et al. (29) found that while in-clinic measures showed a higher effect size compared to daily life gait measures to discriminate cerebellar ataxia from healthy subjects, daily life gait measures were more sensitive to detect longitudinal change over 1 year. These findings show how gait characteristics change across different environmental contexts. While prior studies have largely focused on degenerative cerebellar ataxia broadly, there is limited evidence directly comparing discriminative gait measures in both in-clinic and daily life settings within specific spinocerebellar ataxia subtypes (SCA1, SCA2, SCA3, and SCA6).

The purpose of this study was to identify the gait measures that best discriminate between individuals diagnosed with SCA and age-and sex-matched healthy controls (HC) from a 2-minute walking test at a natural pace in the clinic using wearable inertial sensors. We then compared these prescribed task measures to gait measures collected over a week of free-living activity from daily life. We explored whether the gait measures that are most discriminative between SCA from HC during in-clinic settings are consistent when assessed in daily life. We hypothesized that: (1) distinct gait measures would best discriminate SCA from HC in clinical and daily life settings, and (2) gait characteristics would differ in the same subjects tested in the clinic and daily life.

## Methods

### Participants

Participants were recruited as part of a larger study (IDEA, Instrumented Data Exchange for Ataxia) aimed to examine the gait and balance progression of spinocerebellar ataxia genotypes 1,2,3, and 6 (SCA1-6). Participants enrolled at the OHSU and University of Chicago sites were given the opportunity to participate in 7–14 days of daily life monitoring of their gait quality, immediately following their clinic visits. As part of the larger study's inclusion criteria, participants were limited to those able to walk independently in the clinic, back and forth a 10-meter path for 2 minutes. Exclusion criteria were having a head injury, vestibular dysfunction, stroke, or other neurological condition or musculoskeletal disorder impairing mobility.

### Clinical assessment

All subjects were assessed by a neurologist-specialist using a standardized, validated, eight-domain ratingscale (score range 0–40)—the scale for the assessment and rating of ataxia (SARA), Inventory of Non-Ataxia Signs (INAS), Activities-Specific Balance Confidence Scale (ABC), Patient-Reported Outcome Measure (PROM), and Friedreich's Ataxia Rating Scale—Activities of Daily Living (FARs ADL).

### Clinic gait data collection

In the clinic, data from 3, synchronized, inertial measurement units (IMUs) (Opals by APDM Wearable Technologies- a Clario Company, Portland, OR, USA): one on top of each foot and one over the lower lumbar with an elastic belt were used in this substudy. Each Opal IMU includes a tri-axial accelerometer, gyroscope, and magnetometer with a sampling rate of 128 Hz. Participants completed the 2-minute walk test over a 10-meter pathway as part of a larger battery of tests. The same synchronized sensors and data algorithms were used to derive the same gait measures during the prescribed and daily-life walking.

### Daily-life gait data collection

Immediately after testing in the clinic, subjects were asked to wear instrumented socks (30) on each foot and one Opal sensor over the lower lumbar area with an elastic belt. For daily wear, the Opal IMUs were reconfigured to fit comfortably on each foot within a neoprene wrap, with a battery in a pocket above the lateral malleolus, ensuring the system is easy to use and unobtrusive More details in Shah et al. (30). Subjects were instructed to wear the sensors for at least 8 hours a day for at least 7 days. Data were stored in Opal's internal memory. After 7–14 days of data collection, the socks were returned, and the data were uploaded to a secure database for further processing.

### Measures of gait

In total, 17 gait measures were extracted from the gait in the clinic and daily life. The algorithms for extracting spatial and temporal measures of gait were the same across both clinic and daily life settings, as described and verified in prior studies (31, 32). For daily life gait analysis, the algorithm detects walking bouts using inertial sensor data from the feet and identifies turns based on pelvic yaw rotation (31). Steps are grouped into walking bouts if the interval between steps is less than 2.5 s, and bouts with at least 3 steps lasting at least 3 s are processed using Mobility Lab's commercial algorithms (33–37). The analysis algorithm employs the Unscented Kalman Filter to integrate accelerometer, gyroscope, and magnetometer data, precisely estimating each foot's orientation and trajectory (36, 37). The complete list of measures and definitions is provided in Supplementary Table S1.

Participant gait data were included in the clinic 2-minute walk test if they had at least 25 gait cycles and a total test duration of 110 s or more. For daily life data, inclusion required at least 20 hours of recorded activity over a minimum of 4 days, with at least 20 walking bouts.

### Statistical analysis

Non-parametric statistics were used to evaluate both between-group and within-group differences. Further, the Area Under Curve (AUC) of the Receiver Operating Characteristics (ROC) was used to calculate the between-group discriminatory ability of gait measures. To assess whether gait measures differed by environment, paired Wilcoxon tests were used within each group. To examine the association between gait measures and clinical scores, the Pearson correlation coefficient was used. All statistical analyses were conducted using R software (Version 4.2.0), with statistical significance set at *p* < 0.003 based on Bonferroni's correction (0.05/17 = 0.003, due to 17 measures) to control for multiple comparisons.

## Results

### Demographics and gait activity

This study included 39 people, 26 of whom were diagnosed SCA patients (10 SCA1, 9 SCA2, 4 SCA3, and 3 SCA4) and an additional 13 age and gender similar HC. Age and gender were similar between groups (see Table 1). A total of 3,074 hours of data were collected in daily life, containing 476,477 strides. Activity measures were not different between groups, including stride/hour and turns/hour (see Table 1). The frequency distribution of the median number of strides per bout for the SCA and HC groups is shown in Supplementary Figure S1.

**Table 1:** Demographics and weekly activity of each group.

**Measures**	SCA (*N* = 26)		HC (*N* = 13)		*p*-value
	Median	Q1, Q3	Median	Q1, Q3	
Age (years)	54	38.3, 57	46	32,50	0.1106
Sex (M,F)	13,13	NA	3,10	NA	0.1693
Disease duration (years)	5	3, 10	NA	NA	NA
Total duration (hours)	71.37	58.7,100.8	64.4	56.8,77.1	0.8000
No. of days (#)	7.5	7, 13	7	7, 9	0.3230
Walking bouts/hour (#)	6.4	3.3, 9.8	6.3	3.9, 9.9	0.8000
Strides/hour (#)	124.1	70.7, 220.8	148.4	95.5, 232.6	0.4474
Turns/hour (#)	15.6	6.4, 21.2	16.43	8.7, 20.7	0.8933
No. of strides in a bout (#)	13.8	13, 15.7	15	14, 18	0.0587

*p* Continuous measures compared with Wilxcon Rank Sum Test (Mann–Whitney U). Gender compared using Fisher's Exact Test.

### SCA and HC discriminative ability of clinic vs. daily life gait measures

**Figure 1:**
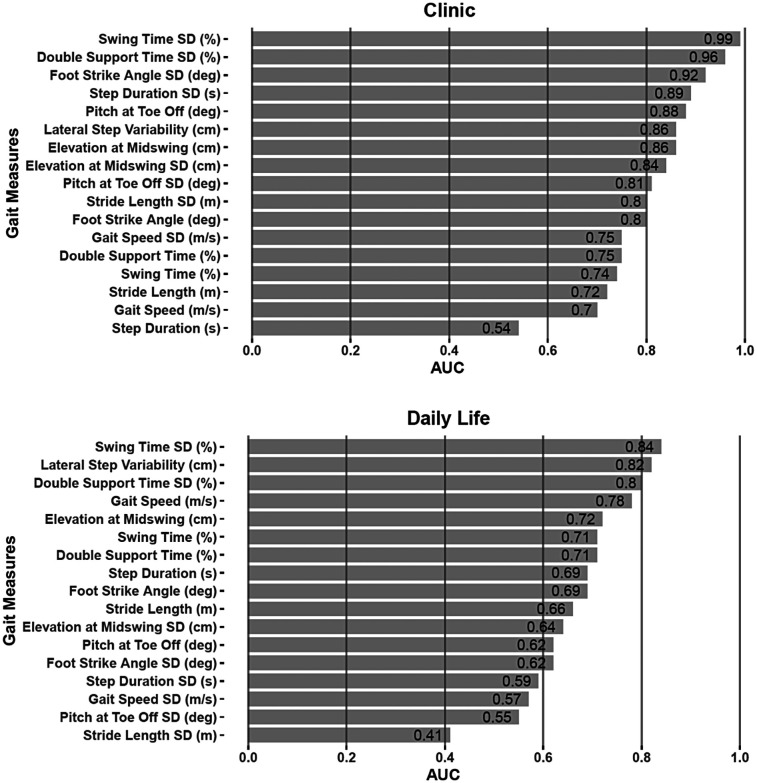
AUC plots distinguishing gait measures of those with SCA vs. healthy controls when measured in the clinic (top) vs. in daily life (bottom).

Measures collected in the clinic consistently outperformed those collected in daily life (Table 2). Overall, there were 10 measures in-clinic, and 3 measures in daily life showed an AUC ≥ 0.8 (Figure 1). Despite the differences in discriminative ability, two measures of gait variability, specifically, the Double Support and Swing Time Standard Deviations (SD), performed strongly in both environments. Double Support Time SD (%) demonstrated an AUC of 0.99 and 0.84 in the clinic and daily life, respectively, while Swing Time SD (%) achieved AUCs of 0.96 and 0.8.

**Table 2:** More gait measures distinguished SCA from healthy control gait in the clinic prescribed 2-minute walk than in daily life walking. Medians and first and third quartiles of gait measures compared between the SCA and HC groups with AUC and Wilcox *p*-values.

Gait measures	Clinic				Daily life			
	SCA (*N* = 26) Median [Q1,Q3]	HC (*N* = 13) Median [Q1,Q3]	AUC [95% CI]	Wilcox *p-*value	SCA (*N* = 26) Median [Q1,Q3]	HC (*N* = 13) Median [Q1,Q3]	AUC [95% CI]	Wilcox *p-*value
Swing time SD (%)	**1.44 [1.05,1.87]**	**0.67 [0.6,0.74]**	**0.99 [0.97–1.00]**	**<0**.**0001**	**2.82 [2.45,3.3]**	**2.08 [1.96,2.32]**	**0.84 [0.71–0.97]**	**0**.**0003**
Double support time SD (%)	**2.16 [1.57,2.71]**	**1.09 [0.97,1.14]**	**0.96 [0.92–1.00]**	**<0**.**0001**	**4.48 [3.85,5.08]**	**3.49 [2.85,3.86]**	**0.80 [0.65–0.95]**	**0**.**0020**
Foot strike angle SD (deg)	**2.32 [1.94,3.33]**	**1.54 [1.31,1.75]**	**0.92 [0.84–1.00]**	**<0**.**0001**	6.74 [5.55,7.47]	7.17 [6.68,7.84]	0.62 [0.43–0.81]	0.2428
Pitch at toe off (deg)	**30.27 [27.32,34.69]**	**37.99 [35.28,39.3]**	**0.88 [0.77–0.99]**	**<0**.**0001**	28.3 [23.81,30.09]	29.46 [28.27,30.68]	0.62 [0.44–0.80]	0.2551
Step duration SD (s)	**0.02 [0.02,0.03]**	**0.01 [0.01,0.01]**	**0.89 [0.80–0.99]**	**<0**.**0001**	0.07 [0.06,0.09]	0.07 [0.06,0.07]	0.59 [0.39–0.80]	0.3683
Elevation at midswing (cm)	**1.83 [1.21,2.37]**	**1.09 [0.68,1.36]**	**0.86 [0.75–0.97]**	**0**.**0003**	4.13 [3.34,4.83]	3.36 [2.92,3.91]	0.72 [0.56–0.88]	0.0272
Lateral step variability (cm)	**4.59 [4.12,5.42]**	**3.32 [2.63,3.52]**	**0.86 [0.74–0.98]**	**0**.**0003**	**7.69 [7.43,8.2]**	**7.07 [6.42,7.27]**	**0.82 [0.68–0.96**]	**0**.**0008**
Elevation at midswing SD (cm)	**0.66 [0.54,0.84]**	**0.47 [0.34,0.54]**	**0.84 [0.71–0.96]**	**0**.**0007**	1.71 [1.36,2.34]	2.1 [1.6,2.45]	0.64 [0.46–0.83]	0.1590
Pitch at toe Off SD (deg)	**2.2 [1.56,3.12]**	**1.35 [1.04,1.93]**	**0.81 [0.67–0.96]**	**0**.**0017**	4.74 [3.77,5.38]	4.38 [4.08,5.17]	0.55 [0.35–0.74]	0.6484
Stride length SD (m)	**0.05 [0.04,0.08]**	**0.04 [0.03,0.04]**	**0.80 [0.66–0.94]**	**0**.**0026**	0.22 [0.18,0.25]	0.22 [0.16,0.23]	0.41 [0.21–0.61]	0.3844
Foot strike angle (deg)	15.95 [12.19,19.66]	20.88 [20.37,21.36]	0.80 [0.65–0.94]	0.0030	19.35 [17.1,24.08]	23.16 [21.52,25.62]	0.69 [0.52–0.86]	0.0578
Gait speed SD (m/s)	0.06 [0.05,0.09]	0.05 [0.04,0.06]	0.75 [0.60–0.91]	0.0110	0.24 [0.21,0.27]	0.26 [0.23,0.3]	0.57 [0.36–0.77]	0.5076
Double support time (%)	21.94 [20.29,25.94]	19.3 [17.82,21.32]	0.75 [0.58–0.91]	0.0126	23.39 [20.41,27.27]	20.07 [18.59,23.34]	0.71 [0.54–0.87]	0.0372
Swing time (%)	39.02 [37.02,39.86]	40.34 [39.33,41.08]	0.74 [0.58–0.91]	0.0146	38.27 [36.33,39.76]	39.96 [38.32,40.72]	0.71 [0.55–0.87]	0.0344
Stride length (m)	1.08 [1,1.21]	1.22 [1.12,1.28]	0.72 [0.57–0.88]	0.0254	1.22 [1.02,1.31]	1.27 [1.2,1.42]	0.66 [0.49–0.84]	0.1050
Gait speed (m/s)	1.00 [0.91,1.23]	1.21 [1.05,1.29]	0.70 [0.54–0.87]	0.0412	1.04 [0.88,1.15]	1.26 [1.1,1.32]	0.78 [0.63–0.93]	0.0043
Step duration (s)	0.53 [0.48,0.56]	0.51 [0.5,0.54]	0.54 [0.36–0.73]	0.6764	0.58 [0.55,0.62]	0.56 [0.53,0.57]	0.69 [0.52–0.86]	0.0578

Bold indicates *p* < .05.

### Gait characteristics differed for the clinic vs. the daily life environments

**Figure 2:**
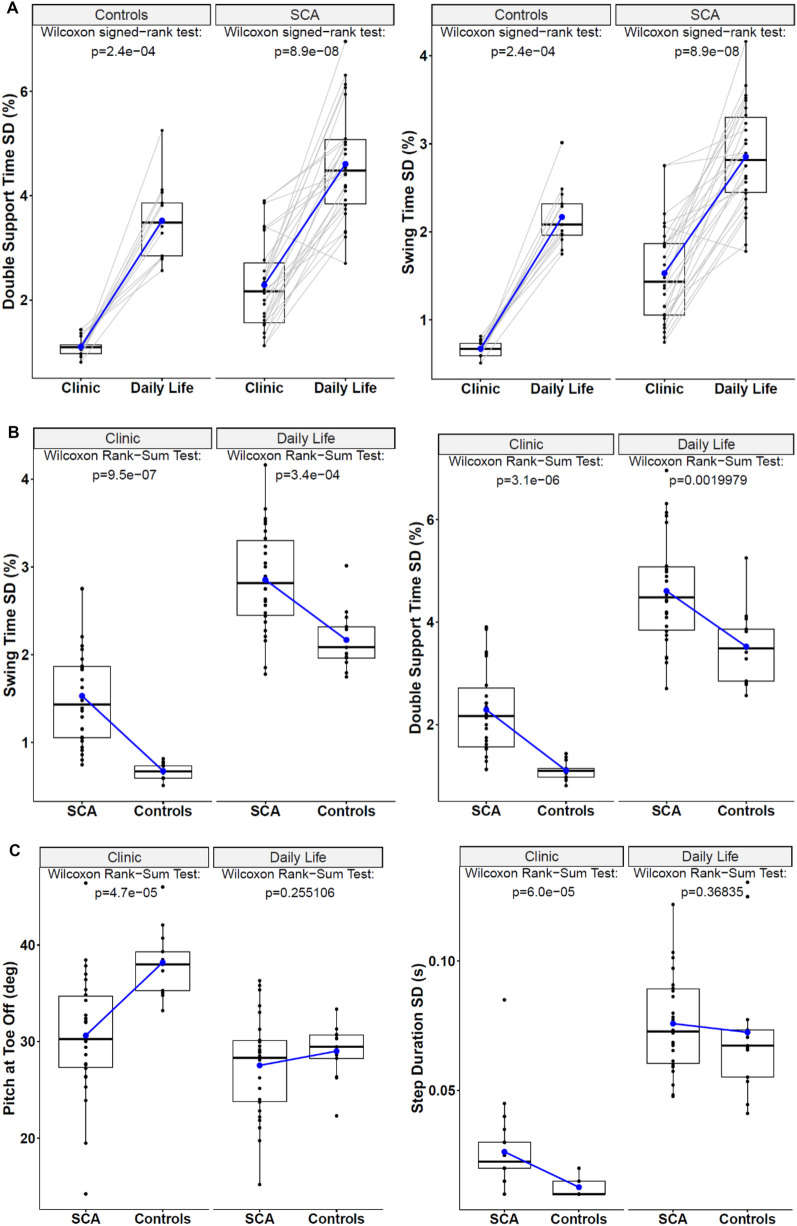
**(A)** Paired box plots demonstrate how the two most discriminative gait measures differed for individual subjects across the clinic vs. daily life environments. **(B)** Box plots of the most discriminative gait measures to compare SCA and HC in both the clinic and daily life environments. **(C)** Examples of Box-Plots for Gait measures that were discriminative to SCA vs. HC in the clinic but not in daily life.

Most (13/17) gait measures significantly differed in the same people with SCA, and 11/17 differed in HC when collected in the clinic vs. daily life (see Supplementary Table S2 and Figure 2A for examples). Although Double Support Time and Swing Time variability were significantly different values when collected in the clinic vs. daily life, both environments showed a statistically significant difference in these gait measures between the SCA and HC cohorts (Figure 2B). Nevertheless, a few measures were significantly different between SCA and HC when collected in the clinic, but not in daily life (Figure 2C).

### Gait measures were significantly correlated with clinical and patient-reported outcomes

**Figure 3:**
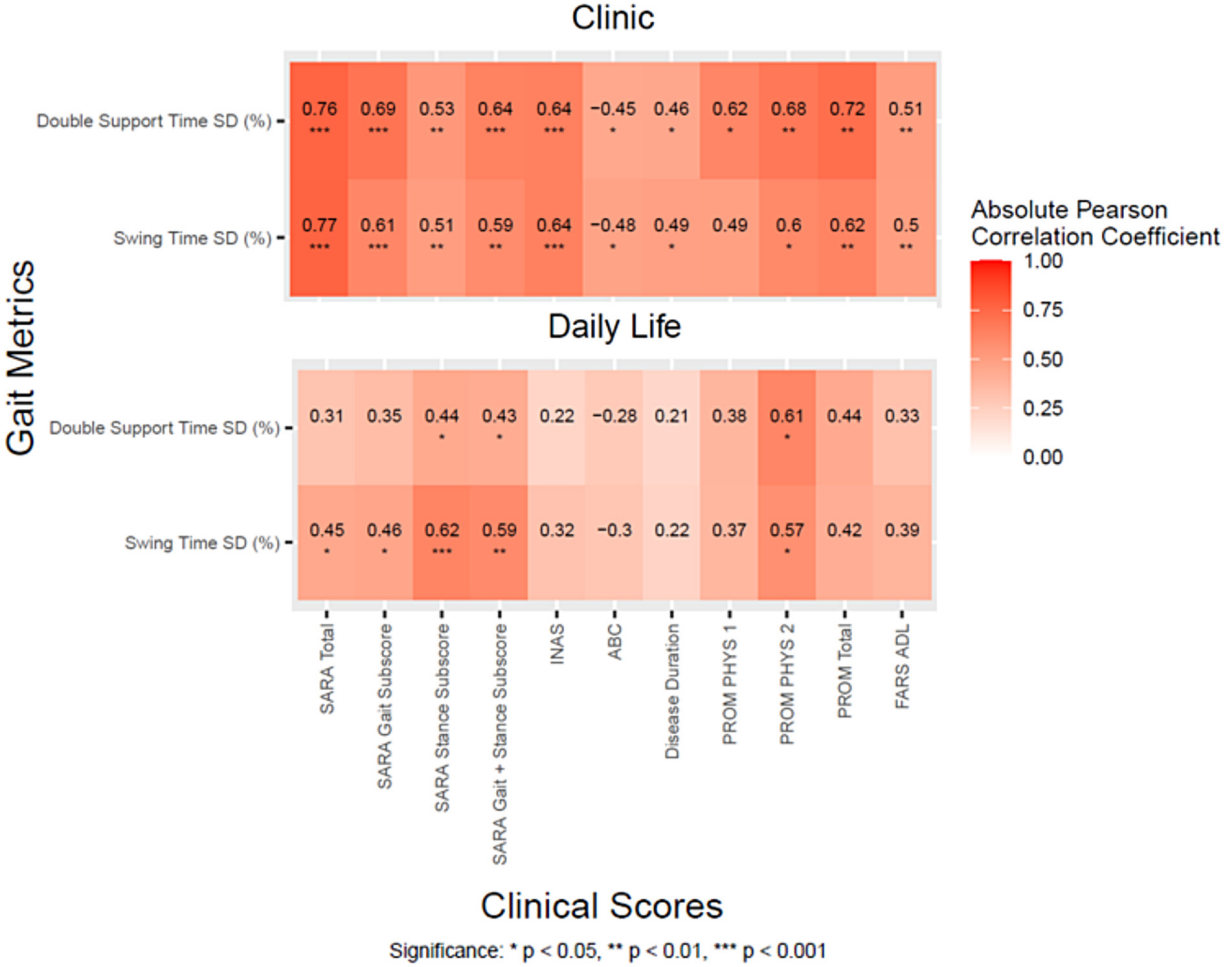
Correlation heatmaps between the two most discriminative gait measures and clinical scores for the SCA cohort during daily life and in the clinic.

The most discriminative variability measures [Double Support Time SD(%) and Swing Time SD (%)] were also significantly correlated with SARA scores and Patient Reported Outcomes such as PROM, ABC, and also Disease Duration in the SCA population (Figure 3). Double support time variability collected in the clinic generally had stronger correlations with clinical measures compared to Double support time variability collected in daily life (i.e., r = 0.76 and 0.77 in the clinic vs. r = 0.31 and 0.45 in daily life with SARA total). The one exception is correlation with the PROM Physical component 2, which was significantly correlated with both discriminative measures in both the clinic and daily life (r = 0.57–0.68).

## Discussion

This study aimed to identify the most discriminatory gait measures for use in clinical trials for SCA 1,2,3, and 6 from body-worn, inertial sensors during a 2-minute, in-clinic walking at natural pace assessment and during a week of walking during daily life. We found that the prescribed walking task in the clinic yielded more discriminative measures than the unprescribed walking in daily life. Yet, two gait timing variability measures were consistently the top discriminative measures for SCA in both settings.

The top three discriminative gait measures between SCA and HC in the clinic and in the home environment were in the variability domain. Swing time SD and Double Support SD were among the top discriminators in both environments, along with foot strike angle SD (clinic) and lateral step variability (daily life). These gait variability measures are consistent with results from previous studies in SCA, showing increased spatiotemporal variability. Shah et al. (16) found that toe-out angle variability and double-support time variability were the most sensitive and specific 2-minute-walk-test gait features of SCA using wearable inertial sensors, with similar findings demonstrated in pre-manifest SCA2 subjects (21). Ilg et al. (11) found that lateral step deviation had an AUC of 0.86 when distinguishing patients with cerebellar ataxia compared to healthy controls in a daily living environment. These findings highlight the clinical significance of gait variability measures collected both during prescribed walking tasks in the clinic and during spontaneous walking in daily living for assessing natural history and intervention studies in ataxia.

Several key differences between in-clinic and home data were observed. First, the clinic 2-minute walk test data showed greater sensitivity to SCA compared to daily life data, as demonstrated by greater AUC values. In fact, 11 out of 17 measures in the clinic and 3 out of 17 measures in daily life had an AUC ≥ 0.8 for discriminating SCA from HC. Second, digital gait measures showed a greater correlation with clinical scales and patient-reported outcomes overall compared to the home environment. This suggests that gait variability measured at home reflects aspects of motor function less aligned with patient perception and clinician-reported performance-related motor assessment.

Gait data captured in a prescribed task in the clinic reflects the patient's capacity to perform gait, whereas data captured in the daily living environment, without task constraints, reflects a patient's actual functional performance in their own environment. The benefits of gait data captured in a clinical task, like the 2-minute walk, include high discriminative ability, correlation with clinical scales, and reliable measurement with relatively quick assessment time. However, patients walk differently in the clinical environment when observed by clinicians and asked to concentrate on their gait in a novel environment (30).

Although variability inherent in passive gait assessment means that more subjects are needed to differentiate ataxic gait from normal, comprehensive daily living gait quality assessment reflects an individual's actual functional mobility in their home and local environment, which provides valuable insights that complement in-clinic assessment. Thus, there are benefits and drawbacks for both in-clinic (e.g., Hawthorne effect, patient burden) (38) and daily living assessment (high variability due to distractions and dual-tasking, large datasets) (39). Results from this study suggest that both in-clinic and daily-living gait variability measures offer utility for clinical trials; however, daily-living assessment may be considered supplemental to a clinic-prescribed gait test, given the limited studies of real-life gait assessment in SCA to date.

There are several limitations to the current study. First, the sample size is limited, with only a few subjects for each of the 4 subtypes of SCA. Future research should aim to gather a larger sample for each subtype of SCA and include additional types, as the discriminative power of gait may vary between SCA subtypes. Second, this study did not take into account other signs of ataxia (ie, upper limb coordination) that could be quantified using body-worn sensors, which may enhance the discriminative power, validity, and reliability for SCA, and allow testing of nonambulatory patients. Future research should explore combining gait and balance measures to develop a composite standing and walking balance score, potentially more sensitive and specific to SCA than a single measure (40). Third, we did not compare similar gait bout lengths between clinical and daily life settings, as we only had data for 2-minute gait bouts in the clinic and observed only a few bouts as long as 2-minute gait bouts in daily life. We have previously shown that people tend to walk faster during a prescribed, self-paced gait test than during daily life when they are distracted and tend to have shorter gait bouts. Forth, due to the small number of participants within each SCA subtype, we were unable to conduct meaningful subtype-specific analyses. Therefore, future studies should include larger cohorts for each subtype to allow more detailed, SCA subtype-specific investigations. Lastly, future studies should include longitudinal progression data to identify which discriminative measures most effectively quantify disease progression.

This study has identified a set of objective and discriminative, digital gait measures from body-worn inertial sensors collected during free living in daily life and during a self-paced, prescribed 2-minute walk in the clinic. The variability of gait timing measures was discriminative for SCA in both daily life and the clinic, but in-clinic measures showed greater discriminative power and higher correlations with clinical scales and patient-reported outcomes. Future research involving tracking disease progression, validity, and reliability of a larger cohort of people with SCA is needed to identify the most useful digital gait biomarkers for clinical trials.

## Data Availability

The datasets presented in this article are not readily available due to patient privacy. De-identified gait and SARA data will be made available with the sponsor and PIs consent. Requests to access the datasets should be directed to cgomez@bsd.uchicago.edu.

## Supplementary material

Supplemental Table S1 Gait measures and their definitions.

Supplemental Table S2 S2A. Paired Wilcoxon results of comparing Clinic versus Daily Life measures for the SCA participants. Bold indicates p<0.003. S2B. Paired Wilcoxon results of comparing Clinic versus Daily Life measures for the HC participants. Bold indicates p<0.003.

Supplemental Figure S1 The frequency distribution of the median number of strides per bout for the SCA and HC groups.
