# Supplementary material for: Clinic vs. daily life gait characteristics in patients with spinocerebellar ataxia
Source: Front Digit Health. 2025 Sep 3;7:1590150. doi: 10.3389/fdgth.2025.1590150 (PMC12440962; doi:10.3389/fdgth.2025.1590150)

Supplementary Table S1.

Gait Measures	Definition
Gait Speed (m/s)	The forward speed of the subject, measured as the forward distance traveled during the gait cycle divided by the stride duration.
Step Duration (s)	The time interval between the initial contact of one foot and the initial contact of the opposite foot.
Stride Length (m)	The forward distance traveled by the foot during a gait cycle.
Double Support (%)	The portion of the stride duration in which both feet are in contact with the ground, calculated as the sum of initial and terminal double support as a percentage of the stride duration.
Swing Time (%)	The portion of the stride duration in which the foot is not in contact with the ground, calculated as the period from the time of toe off until the time of initial contact as a percentage of the stride duration.
Elevation at Mid Swing (cm)	The height of the foot sensor at the moment of maximum forward velocity of the foot, relative to the sensor height during stance.
Pitch at Toe-Off (degrees)	The angle of the foot relative to a level, horizontal surface at the time the foot leaves the ground.
Foot Strike Angle (degrees)	The angle of the foot relative to a level, horizontal surface at the time the foot begins contact with the ground.
Lateral Step Variability (cm)	In a series of 3 consecutive foot placements of the same foot, the variability of perpendicular deviations of the middle foot placement from the line connecting the first and third.

Supplementary Table S2.

S2A.

Gait Measure	Clinic<br>Median [Q1,Q3]	Daily Life<br>Median [Q1,Q3]	*p*-value
**Foot Strike Angle SD (deg)**	**2.3 [2,3]**	**6.7 [6,7]**	**<0.0001**
**Gait Speed SD (m/s)**	**0.1 [0,0]**	**0.2 [0,0]**	**<0.0001**
**Stride Length SD (m)**	**0.1 [0,0]**	**0.2 [0,0]**	**<0.0001**
**Elevation at Midswing SD (cm)**	**0.7 [1,1]**	**1.7 [1,2]**	**<0.0001**
**Lateral Step Variability (cm)**	**4.6 [4,5]**	**7.7 [7,8]**	**<0.0001**
**Double Support Time SD (%)**	**2.2 [2,3]**	**4.5 [4,5]**	**<0.0001**
**Elevation at Midswing (cm)**	**1.8 [1,2]**	**4.1 [3,5]**	**<0.0001**
**Step Duration SD (s)**	**0 [0,0]**	**0.1 [0,0]**	**<0.0001**
**Swing Time SD (%)**	**1.4 [1,2]**	**2.8 [2,3]**	**<0.0001**
**Pitch at Toe Off SD (deg)**	**2.2 [2,3]**	**4.7 [4,5]**	**<0.0001**
**Step Duration (s)**	**0.5 [0,1]**	**0.6 [1,1]**	**<0.0001**
**Foot Strike Angle (deg)**	**15.9 [12,20]**	**19.4 [17,24]**	**0.0001**
**Pitch at Toe Off (deg)**	**30.3 [27,35]**	**28.3 [24,30]**	**0.0020**
Stride Length (m)	1.1 [1,1]	1.2 [1,1]	0.0047
Swing Time (%)	39 [37,40]	38.3 [36,40]	0.4678
Double Support Time (%)	21.9 [20,26]	23.4 [20,27]	0.4992
Gait Speed (m/s)	1 [1,1]	1 [1,1]	0.7644

S2B.

Gait Measure	Clinic<br><br>Median [Q1,Q3]	Daily Life<br><br>Median [Q1,Q3]	*p*-value
**Double Support Time SD (%)**	**1.1 [1,1]**	**3.5 [3,4]**	**0.0002**
**Elevation at Midswing (cm)**	**1.1 [1,1]**	**3.4 [3,4]**	**0.0002**
**Elevation at Midswing SD (cm)**	**0.5 [0,1]**	**2.1 [2,2]**	**0.0002**
**Foot Strike Angle SD (deg)**	**1.5 [1,2]**	**7.2 [7,8]**	**0.0002**
**Gait Speed SD (m/s)**	**0 [0,0]**	**0.3 [0,0]**	**0.0002**
**Lateral Step Variability (cm)**	**3.3 [3,4]**	**7.1 [6,7]**	**0.0002**
**Pitch at Toe Off (deg)**	**38 [35,39]**	**29.5 [28,31]**	**0.0002**
**Pitch at Toe Off SD (deg)**	**1.4 [1,2]**	**4.4 [4,5]**	**0.0002**
**Step Duration SD (s)**	**0 [0,0]**	**0.1 [0,0]**	**0.0002**
**Stride Length SD (m)**	**0 [0,0]**	**0.2 [0,0]**	**0.0002**
**Swing Time SD (%)**	**0.7 [1,1]**	**2.1 [2,2]**	**0.0002**
Step Duration (s)	0.5 [0,1]	0.6 [1,1]	0.0046
Foot Strike Angle (deg)	20.9 [20,21]	23.2 [22,26]	0.0134
Stride Length (m)	1.2 [1,1]	1.3 [1,1]	0.0134
Double Support Time (%)	19.3 [18,21]	20.1 [19,23]	0.0681
Swing Time (%)	40.3 [39,41]	40 [38,41]	0.0803
Gait Speed (m/s)	1.2 [1,1]	1.3 [1,1]	0.2439

Supplementary Figure S1

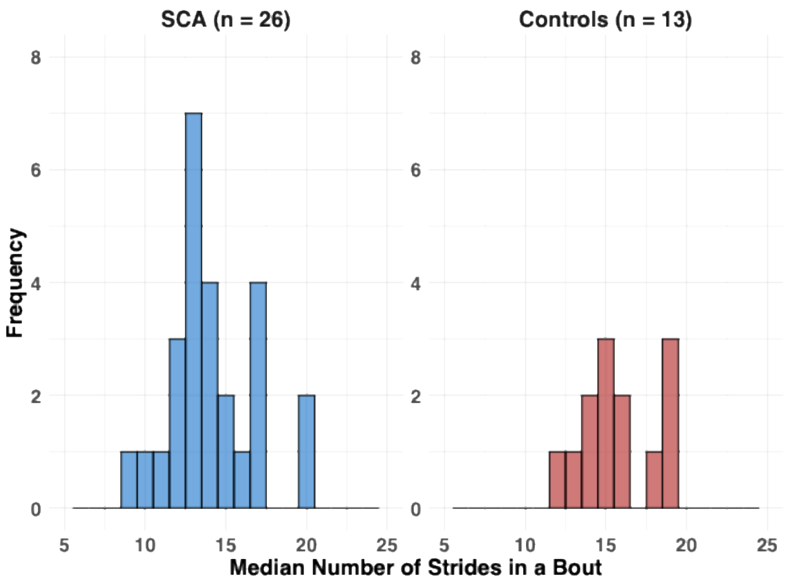

Supplement: Supplemental Figure S1 — The frequency distribution of the median number of strides per bout for the SCA and HC groups. [file Datasheet1.pdf]
